# Sustainable Development Goals and microplastics in seafood: a theoretical-reflective study

**DOI:** 10.1590/0034-7167-2024-0408

**Published:** 2025-09-01

**Authors:** Pedro Henrique Santos de Lima, Renata Gomes Barreto, Cícera Patrícia Daniel Montenegro, Andressa Fernanda Silva, Fábio de Souza Terra, Murilo César do Nascimento, Mônica Lá Salette da Costa Godinho, Maria Lucia do Carmo Cruz Robazzi

**Affiliations:** I Universidade Federal da Paraíba. João Pessoa, Paraíba, Brazil; II Universidade de São Paulo. Ribeirão Preto, São Paulo, Brazil; III Universidade Federal de Alfenas. Alfenas, Minas Gerais, Brazil

**Keywords:** Microplastics, Human Health, Foods, Seafoods, Health Personnel., Microplásticos, Salud Humana, Alimentos, Alimentos Marinos, Personal de Salud.

## Abstract

**Objectives:**

to analyze the scientific literature on the contamination of marine animals by plastic microparticles and identify problems caused by human consumption of these foods.

**Methods:**

a theoretical-reflective study, prepared through a narrative review. To acquire the texts for the study, keywords were searched in the Health Sciences Descriptors, and the search was carried out in information sources, with publications between 2017-2024.

**Results:**

there is possible human contamination by seafood, which is consumed “in natura” and/or canned-packaged, with the detected presence of plastic microparticles, which can accumulate in the human body.

**Final Considerations:**

plastic particles in seafood can cause harm to health. Public policies aimed at preventing water pollution should be strengthened, as well as providing guidance to health personnel on the problems that foods with plastic microparticles can cause, in order to be able to be closer to the action targets of the Sustainable Development Goals.

## RESUMEN

**Objetivos:**

analizar la literatura científica sobre la contaminación de animales marinos por micropartículas plásticas e identificar problemas causados por el consumo humano de estos alimentos.

**Métodos:**

estudio teórico-reflexivo, elaborado a través de una revisión narrativa. Para adquirir textos para el estudio se buscaron palabras clave en Descriptores de Ciencias de la Salud, y la búsqueda se realizó en fuentes de información, con publicaciones entre 2017-2024.

**Resultados:**

existe posible contaminación humana por alimentos marinos, que se consumen “frescos” y/o envasados enlatados, detectándose presencia de micropartículas plásticas, que pueden acumularse en el cuerpo humano.

**Consideraciones Finales:**

las partículas de plástico en los alimentos marinos pueden perjudicar la salud. Se deben reforzar las políticas públicas encaminadas a prevenir la contaminación del agua, así como orientar al personal de salud sobre los problemas que pueden causar los alimentos con micropartículas de plástico, para poder acercarse a las metas de acción de los Objetivos de Desarrollo Sostenible.

## INTRODUCTION

The Sustainable Development Goals (SDGs) were established by the United Nations in 2015 and comprise an action plan committed to people, the planet and prosperity, consisting of 17 goals and 169 interconnected global action targets to be achieved by 2030, comprehensively addressing the main development challenges worldwide. Countries should then define and develop their national goals and incorporate these goals and actions into their work plans and programs, and into their government and state policies. To achieve the SDGs, it was necessary for governments, civil society, the private sector and all people to work together, pooling efforts and actions aimed at achieving the goals and targets, to build a fair, prosperous and sustainable planet for all generations^(1)^.

Regarding water and soil pollution/contamination, which the SDGs aim to minimize, it is necessary to reflect on plastic pollution, since this material has become very common in people’s daily lives. Plastics are materials that have been used in most countries for years and this use has caused many consequences, as they have been found in food, cosmetics, water, air, soil, vegetables, various products, and in the bodies of animals and humans, among others. They are considered complex and heterogeneous synthetic chemical substances, and more than 98% are produced from fossil carbon (coal, oil, and gas)^(2)^.

Between 1950 and 2017, approximately 9.2 billion tons of plastic were produced. In 2021, production reached 400 million tons^(3)^ and estimates are that by 2050, global annual production will exceed 1.1 billion tons/year^(4)^. Processing companies need it for their production; however, its disposal has been done inappropriately, damaging animal and plant life and that existing in ocean waters^(5)^.

Through various processes, including biodegradation, these plastics are transformed into particulate matter, almost invisible, ranging from 5 millimeters (mm) to 100 nanometers (nm). In contact with water, these particles are usually found in reefs and corals^(6)^, and are ingested by smaller organisms such as plankton, crustaceans and shellfish^(7)^ and by other marine animals, and, consequently, are being identified in humans who consume them^(8)^. In the human body, in laboratory tests, microplastics have been shown to cause damage to human cells, including allergic reactions and cell death. They have been found in the blood of healthy donors and in lung samples taken from patients, causing lung irritation, dizziness, headaches, asthma and cancer, among other health hazards^(9)^.

In this context, the following question was raised: is there information describing the contamination of particulate plastic material in foods from the sea and consumed by humans? This theoretical and reflective study was then developed through a narrative literature review to identify the presence of plastic materials in seafood.

## OBJECTIVES

To analyze scientific literature on the contamination of marine animals by plastic microparticles and to identify problems caused by human consumption of these foods.

## METHODS

This is a theoretical-reflective study, prepared through a literature review, in narrative form, considered a broad publication, appropriate for describing and discussing the development of a given subject, from a theoretical or contextual point of view. In general, it does not need to identify the sources of information used, nor the methodology for searching for such sources of information, nor the criteria used in the assessment and selection of works. It consists of an analysis of literature published in scientific publications, in the interpretation and critical analysis of the authors, which allows readers to obtain and update knowledge on a specific topic in a short space of time, although it does not have a methodology that allows data reproduction^(10)^.

For this study, the terms in Portuguese (*Microplásticos, Saúde Humana, Alimentos, Alimentos Marinhos*) and the corresponding versions in English (Microplastics, Human Health, Foods, Seafoods) and Spanish (*Microplásticos, Salud Humana, Alimentos, Alimentos Marinos*) were searched in the Health Sciences Descriptors (DeCS) between 2017 and 2024. Thus, the theoretical-reflective text was composed after consulting the sources Embase, Scopus, Medline/PubMed, Web of Science, CINAHL (EBSCO), LILACS, BDENF, ProQuest Dissertations & Theses Citation Index and Google Scholar, covering the aforementioned period. During the search for materials, 129 scientific texts were identified, all international, that addressed the presence of plastic particulate matter in various types of food. In the present study, the authors emphasized human contamination by seafood, which can be consumed “*in natura*” and/or canned-packaged, containing plastic microparticles (PM).

## RESULTS

Subsequently, of the 129 studies found, 23 addressed the human consumption of marine animals with plastic microparticles and are presented with letters ranging from A to X (Chart 1).

**Chart 1:** Scientific texts involving the topic of seafood contaminated by microplastic particles and consumed by humans. 2017-2024 (n=23)

Groups	Identification/title	Authors	Country/year/ source of information	Summary	Study design
**MOLLUSCS**	A. The observation of starch digestion in blue mussel Mytilus galloprovincialis exposed to microplastic particles under varied food conditions	O’Brien CJ, Hong HC, Bryant EE, Connor KM	United States 2021 PubMed	The impact of microplastics (PM) on the amylase enzyme activity of *Mytilus galloprovincialis* was investigated under controlled laboratory conditions. It was found that exposure to extreme levels of PM increased amylase activity; however, these mussels may be resilient to current levels of PM pollution in nature.	Experimental
	B. An overview of microplastics in oysters: Analysis, hazards, and depuration	Liu Y, Shi H, Chen L, Teng X, Xue C, Li Z.	China 2023 PubMed	The impact of PMs on marine ecosystems, especially oysters, was discussed and the need for standardized analytical methods for their quantification in shellfish was highlighted.	Review
	C. The impact of nano/micro-plastics toxicity on seafood quality and human health: facts and gaps	Gündogdu, S, Rathod N, Hassoun A, Jamroz E, Kulawik P, Gokbulut C, Özogul F.	Peru India Poland France 2023 PubMed	Environmental pollution and the resulting contamination of seafood with PM pose a potential threat to consumers, with the greatest risk associated with algae, salt, shellfish and small fish.	Review
**CRUSTACEANS**	D. Estimation of contamination level in microplastic-exposed crayfish by laser confocal micro-Raman imaging	Xiao X, Liu X, Mei T, Xu M, Lu Z, Dai H, *et al*.	China 2023 PubMed	The levels of PM contamination in crayfish exposed to different PM concentrations and exposure durations were demonstrated using Laser Confocal Micro-Raman (LCM-Raman) imaging and scanning electron microscopy techniques. LCM-Raman imaging combined with image processing technology can be used to build a high-performance analytical strategy for the assessment of microplastic contamination in crayfish.	Experimental
	E. Microplastics in feed cause sublethal changes in the intestinal microbiota and a non-specific immune response indicator of the freshwater crayfish Procambarus clarkii (Decapoda: Cambaridae)	Guillén-Watson R, Arias-Andres M, Rojas-Jimenez K, Wehrtmann IS.	Costa Rica 2023 PubMed	PMs are hazardous pollutants of global concern that threaten aquatic ecosystems and public health and alter the gut microbial composition of *P. clarkii*. Exposure to PMs reduced the abundance of *Clostridia* and *Bateroidetes*, which are important for immune system development and pathogen prevention.	Experimental
	F. Progress on the Effects of Microplastics on Aquatic Crustaceans: A Review	Zhang S, Wu H, Hou J.	China 2023 PubMed	The effects of PMs on aquatic crustaceans were understood, providing a basic framework for the ecological threat of PMs to these crustaceans.	Review
	G. Microplastic-mediated transport of PCBs? A depuration study with Daphnia magna	Gerdes Z, Ogonowski M, Nybom I, Ek C, Adolfsson-Erici M, Barth A, *et al*.	Sweden 2019 EMBASE	The effect of PM on the removal of polychlorinated biphenyls (PCBs) from planktonic animals was assessed by exposing the cladoceran *Daphnia magna* with a high body burden of PCBs (18, 40, 128 and 209) to a mixture of microplastics and algae, and daphnia exposed only to algae served as controls. There was a higher production of size-specific eggs in animals carrying PCBs and receiving food mixed with PM. However, the effects of PM exposure on fecundity were of low biological significance, since the PCB body burden and exposure concentrations were much higher than the relevant environmental concentrations.	Experimental
	H. Can microplastics influence the accumulation of pb in tissues of blue crab?	Munuera P, Salvat-Leal I, Belmonte A, Romero D	Spain 2021 PubMed	The distribution and accumulation of Pb in the tissues of blue crabs (*Callinectes sapidus*) exposed to PM was assessed. The presence of PM in the water did not appear to increase the accumulation of Pb in the tissues of blue crabs.	Experimental
**FISH**	I. Reduced cellular process and developmental process genotoxicity of polystyrene nanoplastics in zebrafish embryogenesis using Aurelia aurita proteins	Park IH, Geum SW, Yeo M-K.	Korea 2023 PubMed	Zebrafish embryos were exposed to polystyrene nanoplastics (PS-NP), mucin (Mu), and Mu + PS-NP to assess their effects on genotoxicity. Based on the phenotypic abnormalities in juvenile fish, mRNA sequencing was used to determine the changes in gene transcription levels induced by PS-NP. PS-NP induced changes in mRNA expression and had toxic effects causing phenotypic abnormalities in zebrafish embryos.	Experimental
	J. Assessment of microplastic contamination in an eastern Pacific tuna (Katsuwonus pelamis) and evaluation of its health risk implication through molecular docking and metabolomics studies	Wu L, Dai X, Xu J, Ou D, Wang L, Lin H, *et al*	China 2023 PubMed	The PM pollution in a commercially important tuna species, *Katsuwonus pelamis* from the Eastern Pacific, and its health implications were investigated. The presence of PM in various parts of the fish was analyzed, identifying the predominant types of PM such as polyester and polyethylene terephthalate (PET) and examining their molecular interactions with glycerol protein kinase.	Experimental
	K. Natural history matters: Plastics in estuarine fish and sediments at the mouth of an urban watershed	Talley TS, Venuti N, Whelan R.	United States 2020 EMBASE	A section of Chollas Creek in San Diego, California, was examined to determine: the extent and magnitude of microplastic pollution in estuarine sediments and fish; the extent and magnitude of Semi-Volatile Organic Compound (SVOC) contamination in fish; and whether they ingested which types of PM compared to the PM composition of streambed sediments. The sediments (0-5 cm depth) contained approximately 10,000 small pieces of plastic per m^2^, consisting of 90% fibers and hard and soft pieces. Almost 25% of fish contained small plastics; prevalence varied with size and between species; of the 25 types of plastics found in the sediments, fish preferred approximately 10 types; several SVOCs, both water-soluble and sediment-associated compounds, were found in both fish species tested.	Experimental
	L. Presence of microplastics in commercial canned tuna	Diaz-Basantes MF, Nacimba-Aguirre D, Conesa JA, Fullana A	Ecuador Spain 2022 Elsevier	The presence of synthetic polymeric microparticles was determined in canned tuna samples. The identification of PM by fluorescence and FTIR microspectrometry revealed that PET, polystyrene and nylon were the most present in the analyzed samples.	Experimental
	M. No evidence of spherical microplastics (10-300 μm) translocation in adult rainbow trout (Oncorhynchus mykiss) after a two-week dietary exposure	Kim J, Poirier D. G, Helm P. A, Bayoumi M, Rochman C. M.	Canada 2020 PubMed	In trout, PMs have been found to be translocated from the intestine to other tissues, making exposure from eating fish fillets or other seafood products a potential concern. However, no particles were found in the fillets, liver, or gonads of the fish, suggesting that translocation of spherical PMs did not occur in adult rainbow trout.	Experimental
	N. The effect of environmental stressors on growth in fish and its endocrine control	Canosa LF, Bertucci JI.	Argentina Spain 2023 PubMed	An overview and update on the effects of various abiotic anthropogenic factors on growth hormone and food intake regulation was obtained. PM can be ingested by fish and accumulate in the stomach, generating a feeling of satiety or causing damage that can inhibit food intake. Their accumulation in the stomach of fish can alter the response of peripheral factors that regulate food intake.	Review
**SEAFOOD**	O. Bioaccumulation and biomagnification of microplastics in marine organisms: A review and meta-analysis of current data	Miller ME, Hamann M, Kroon FJ.	Australia 2020 PubMed	Contamination by PMs in the marine environment is a growing problem. Previous studies have shown that PMs bioaccumulate in marine organisms, but the biomagnification of these compounds along the food web is still debated. This systematic literature review found no clear evidence of PM biomagnification in a general marine food web, although PM bioaccumulation occurs within trophic levels. The lack of data on trophic transfer and biomagnification of chemical additives associated with PMs is a gap that needs to be filled in the future.	Systematic review with meta-analysis
**SEAFOOD**	P. Microplastics in seafood: How much are people eating?	Wendee Nicole	United States 2021 PubMed	The presence of PMs in several marine species, including fish and shellfish, has been quantified, indicating that these particles are widely distributed in the marine environment. Regular consumption of seafood can lead to cumulative ingestion of PMs, raising concerns about potential long-term health effects.	Theoretical-reflective
	Q. Microplastics as a new, ubiquitous pollutant: Strategies to anticipate management and advise seafood consumers	Susan E. Farady	United States 2019 Science Direct	The presence of PM in the oceans is increasing, affecting marine life and potentially human health through the consumption of seafood. Its ingestion by marine organisms can have adverse impacts on consumers’ health. It is suggested that strategies be implemented to manage and mitigate PM pollution, including reduction policies and consumer awareness.	Theoretical-reflective
	R. - Investigating microplastics bioaccumulation and biomagnification in seafood from the Persian Gulf: a threat to human health?	Razegheh Akhbarizadeh, Farid Moore, Behnam Keshavarzi	Iran 2019 PubMed	The presence of PMs in five commercial seafood species was investigated. PMs were present in all specimens, with higher levels found in muscles and gills of burrowing and filter-feeding species. However, no evidence of PM biomagnification was found in the edible parts of seafood, suggesting that trophic dilution rather than magnification occurs. Therefore, it is important to monitor seafood consumption for vulnerable groups, such as pregnant or lactating women and their children, to ensure their safety.	Experimental
	S. Microplastics in fisheries and aquaculture: status of knowledge on their occurrence and implications for aquatic organisms and food safety	Lusher A, Hollman P, Mendoza-Hill J	United Kingdom Netherlands Spain Italy 2017 PubMed	Increased plastic production has led to significant contamination of aquatic environments. PMs can be ingested by aquatic organisms and accumulate toxic chemicals. However, studies have shown that human ingestion of microplastics is reduced by removal from the gastrointestinal tract in most species of seafood consumed. Furthermore, exposure to microplastics after seafood consumption is considered negligible.	Review
	T. Microplastic contamination of seafood intended for human consumption: A systematic review and meta-analysis	Danopoulos E, Jenner LC, Twiddy M, Rotchell JM.	United Kingdom 2020 PubMed	A systematic review and meta-analysis of PM contamination levels in seafood was conducted and annual human uptake was subsequently estimated. Microplastics were found in seafood samples that constitute important vectors for human exposure to PM.	Systematic review with meta-analysis
	U. Vibrio parahaemolyticus and Vibrio vulnificus in vitro biofilm dispersal from microplastics influenced by simulated human environment	Leighton RE, Xiong L, Anderson GK, Astarita GM, Cai G, Norman RS, *et al*	United States 2023 PubMed	Ingestion of microplastics contaminated with Vibrio biofilms may pose a risk to human health. A study investigated how different environmental conditions affect the dispersal of Vibrio parahaemolyticus and Vibrio vulnificus biofilms on PM. The results showed that both species can disperse rapidly on different types of plastics, but the dispersal process is highly variable and dependent on the type of plastic and environmental conditions. This suggests that ingestion of contaminated PM may pose a risk to human health, especially in areas with high concentrations of PM.	Experimental
	V. Microplastics: A Real Global Threat for Environment and Food Safety: A State of the Art Review	Ziani K, Ioniţă-Mîndrican C-B, Mititelu M, Neacşu SM, Negrei C, Moroşan E, *et al.*	Romania 2023 PubMed	The widespread presence of PM in the environment and its impact on wildlife and human health were analyzed. Their detection in various marine species, drinking water and common foods such as salt, honey and marine organisms was discussed.	Review
	X. Uncovering microplastics contamination in canned seafood	Silva DM, Almeida CMR, Guardiola FA, Pereira R, Rodrigues SM, Ramos S.	Portugal and Spain 2024 Elsevier	Sixty cans of seafood (sardines in tomato sauce, in sunflower oil, octopus in tomato sauce, mussels in escabeche sauce, tuna in olive oil and mackerel in sunflower oil) from four popular brands from Portugal and Spain were investigated. All types of canned seafood investigated were contaminated with PM.	Experimental

## DISCUSSION

The researchers who conducted these studies were European, North American, Asian, Oceanian, Central American and South American, demonstrating the global problem of PM in marine animals consumed by humans. The language of all studies was English; most involved North American researchers; the source of information in which they were found was PubMed; most studies were not conducted in isolation, but by groups of researchers and some involved scholars from several countries simultaneously.

It has become clear that global pollution of particulate matter in the seas is a serious problem, which contradicts the data projected by the UN in relation to the 2030 Agenda SDGs. In other words, by 2030, in relation to health and well-being, a healthy life should be ensured and well-being should be promoted for everyone at all ages. Moreover, there would need to be a reduction in environmental pollution/contamination, and countries would need to reinforce risk reduction and management of national and global health risks, as recommended in the 2030 Agenda cited above. However, on the contrary, water, soil and air are polluted and causing severe disorders in living beings. It is also strange that healthcare professionals are so incipient in their work in relation to this problem, as there seems to be a certain lack of knowledge on the subject, although there is important research on the subject, as shown in the texts analyzed in this review.

Regarding contamination of seafood, PMs were found in mollusks such as mussels, oysters, algae, shellfish and other small fish (texts A-C). In Brazil, it is estimated that marine organisms are ingesting plastics, including zooplankton, fish and turtles, mammals and seabirds, many of which are already threatened with extinction. These marine species are coming into contact with waste from human production and are also dying because of it^(11)^.

In relation to crustaceans, contamination of crayfish (texts D-E) and lead in crabs (text H) consumed by humans were identified. In Brazil, it was identified how much pollution affected mangrove crabs’ lives in coastal cities, subjected to the large quantity of heavy metals found in the water and ingested by Brazilians, putting these consumers’ health at risk^(12)^.

As for fish, several studies have shown that pollution by polystyrene PM was genotoxic in zebrafish embryos in Korea (text I), contaminating tuna in China (text J), and was also found by Spanish and Ecuadorian researchers in commonly marketed tuna (article l). In seafood, it was also shown to be a contaminant in shellfish and fish, indicating that this particulate matter can cause long-term health damage (texts P, Q) in studies conducted in the Persian Gulf (text R) as well as in other Asian and European countries. Canned seafood also presents significant contamination, as found in Portugal and Spain, in all cans examined (text “X”).

It is noted that despite Brazil having a significant coastline, possibly much larger than many of the countries identified in this reflective text, no studies on this topic were found, especially during the period investigated, nor was the presence of healthcare professionals in this context found. Failures in the waste management system have been blamed for the problem of plastic pollution. Blame usually centers on consumers (who dispose of these materials inappropriately) and on cities, which lack selective collection and recycling systems. Political solutions have focused on improving the recyclability and recycling rates of plastic products and packaging^(11)^.

In the context of seeking to achieve sustainable development, healthcare professionals should play a vital role in achieving these goals and objectives. SDG 3 is dedicated to “Good health and well-being” and, in its composition, there are nine extremely complex and challenging goals for the whole of society. The goal of reducing the number of deaths and illnesses caused by hazardous chemicals, contamination and pollution of air and water and soil^(13)^ stands out here, which would require the involvement of more assertive actions by everyone, including those in the health sector.

## FINAL CONSIDERATIONS

Based on the findings of this study, the presentation of scientific texts and the reflections made, the relationship between human exposure and contamination by PMs present in seafood consumed by the population and which can cause harm to health is highlighted. Future studies are needed to demonstrate, in greater depth, the damage that such PMs can cause to people’s bodies. Therefore, it is important to create a greater number of national public policies aimed at preventing water pollution, as well as to provide guidance to health teams on the damage that foods with PMs can cause, in order to bring the country closer to the SDG action targets.

It is hoped that this text can alert healthcare professionals to provide care and guidance to users of healthcare services on this topic.
