# Supplementary material for: Sustainable Development Goals and microplastics in seafood: a theoretical-reflective study
Source: Rev Bras Enferm. 2025 Sep 1;78(Suppl 1):e20240408. doi: 10.1590/0034-7167-2024-0408 (PMC12407883; doi:10.1590/0034-7167-2024-0408)
Supplement: Translation

# Translation: Objetivos do Desenvolvimento Sustentável e microplásticos em alimentos marinhos: estudo teórico-reflexivo

**DOI:** 10.1590/0034-7167-2024-0408pt

**Authors:** Pedro Henrique Santos de Lima, Renata Gomes Barreto, Cícera Patrícia Daniel Montenegro, Andressa Fernanda Silva, Fábio de Souza Terra, Murilo César do Nascimento, Mônica Lá Salette da Costa Godinho, Maria Lucia do Carmo Cruz Robazzi

**Affiliations:** I Universidade Federal da Paraíba. João Pessoa, Paraíba, Brasil; II Universidade de São Paulo. Ribeirão Preto, São Paulo, Brasil; III Universidade Federal de Alfenas. Alfenas, Minas Gerais, Brasil

**Keywords:** Microplásticos, Saúde Humana, Alimentos, Alimentos Marinhos, Pessoal de Saúde.

## Abstract

**Objetivos:**

analisar a literatura científica sobre a contaminação de animais marinhos por micropartículas plásticas e identificar problemas acontecidos pelo consumo humano destes alimentos.

**Métodos:**

estudo teórico-reflexivo, elaborado por uma revisão narrativa. Para aquisição dos textos para o estudo foram pesquisadas palavras-chave nos Descritores de Ciências da Saúde e a busca foi realizada em fontes de informações, com publicações entre 2017-2024.

**Resultados:**

há uma eventual contaminação humana pelos alimentos marinhos, que são consumidos “*in natura*” e/ou enlatados-embalados, com a presença detectada de micropartículas plásticas, que podem se acumular no organismo humano.

**Considerações Finais:**

partículas plásticas em alimentos marinhos podem trazer danos à saúde; devem ser reforçadas políticas públicas direcionadas à prevenção da poluição das águas, assim como, proporcionar a orientação do pessoal de saúde sobre os problemas que alimentos com micropartículas de plástico podem causar, para ser possível ter-se uma maior aproximação às metas de ação dos Objetivos de Desenvolvimento Sustentável.

## INTRODUÇÃO

Os Objetivos de Desenvolvimento Sustentável (ODS) foram estabelecidos pela Organização das Nações Unidas em 2015 e compõem um plano de ação comprometido com as pessoas, o planeta e a prosperidade, constituído por 17 objetivos e 169 metas de ação global interconectadas, a serem alcançadas até 2030 abordando, de forma integral, os principais desafios de desenvolvimento em todo o mundo. Os países deveriam, então, definir e desenvolver suas metas nacionais e incorporar tais objetivos e ações em seus planos e programas de trabalho, nas suas políticas de governo e de estado. Para o alcance dos ODS, tornava-se necessário que governos, sociedade civil, setor privado e todos as pessoas trabalhassem em conjunto, somando esforços e ações direcionadas ao alcance dos objetivos e metas, para a construção de um planeta justo, próspero e sustentável para todas as gerações^(1)^.

No que se refere à poluição/contaminação da água e do solo, prevista nos ODS para ser minimizada, torna-se necessário refletir sobre a poluição plástica, visto que esse material tornou-se muito comum no cotidiano de vida das pessoas. Plásticos são materiais que têm sido utilizados, na maioria dos países há anos e este uso tem provocado muitas consequências, já que têm sido encontrados em alimentos, em cosméticos, na água, no ar, no solo, em vegetais, em produtos variados, em corpos de animais e de humanos, entre outros. São considerados substâncias químicas sintéticas complexas e heterogêneas; mais de 98% são produzidos a partir de carbono fóssil (carvão, petróleo e gás)^(2)^.

Entre os anos de 1950-2017, foram produzidos cerca de 9,2 bilhões de toneladas de plástico; em 2021, a produção chegou a 400 milhões de toneladas^(3)^ e as estimativas são que até 2050 a produção anual global excederá 1,1 bilhão de toneladas/ano^(4)^. Empresas processadoras precisam dele para sua produção; entretanto, o seu descarte tem sido feito de forma inadequada, danificando a vida animal, vegetal e a existente em águas oceânicas^(5)^.

Por meio de vários processos, incluindo a biodegradação, esses plásticos transformam-se em material particulado, quase invisível, correspondendo de 5 milímetros (mm) a 100 nanômetros (nm). Em contato com águas, estas partículas costumam ser encontradas em recifes e corais^(6)^ e são ingeridas por organismos menores como plâncton, crustáceos e mariscos^(7)^ e pelos demais animais marinhos e, consequentemente, estão sendo identificadas no ser humano que os consome^(8)^. No corpo humano, em testes de laboratório, os microplásticos demonstraram causar danos às células humanas, incluindo reações alérgicas e morte celular; foram encontrados em sangues de doadores saudáveis e em amostras pulmonares retiradas de pacientes, ocasionando irritação pulmonar, tontura, dores de cabeça, asma e câncer, entre outros malefícios à saúde^(9)^.

Mediante este contexto, questionou-se: existem informações descrevendo a contaminação de material plástico particulado nos alimentos provenientes de mares e consumido pelo ser humano? Elaborou-se, então, o presente estudo teórico reflexivo, por meio de revisão da literatura de forma narrativa, para identificar a presença de materiais plásticos em alimentos marinhos.

## OBJETIVOS

Analisar a literatura científica sobre a contaminação de animais marinhos por micropartículas plásticas e identificar problemas acontecidos pelo consumo humano destes alimentos.

## MÉTODOS

Estudo teórico-reflexivo, elaborado por meio de uma revisão da literatura, de forma narrativa, considerada uma publicação ampla, apropriada para descrever e discutir o desenvolvimento de um determinado assunto, sob ponto de vista teórico ou contextual. Em geral não necessita identificar as fontes de informação utilizadas, nem a metodologia para a busca de tais fontes de informação, nem os critérios utilizados na avaliação e na seleção dos trabalhos. Constitui-se de uma análise da literatura veiculada em publicações científicas, na interpretação e análise crítica dos autores, que permite ao leitor obter e atualizar o conhecimento sobre uma temática específica em curto espaço de tempo, embora não possua metodologia que permita a reprodução dos dados^(10)^.

Para este estudo, foram pesquisados, entre 2017-2024, nos Descritores de Ciências da Saúde (DECS) os termos em português (Microplásticos, Saúde Humana, Alimentos, Alimentos Marinhos) e as correspondentes versões em inglês (*Microplastics, Human Health, Foods, Seafoods*) e espanhol (*Microplásticos, Salud Humana, Alimentos, Alimentos Marinos*). Assim, o texto teórico-reflexivo foi constituído após a consulta nas fontes de informações: Embase, Scopus, Medline/PubMed, *Web of Science*, CINAHL (EBSCO), LILACS, BDENF, *ProQuest Dissertations & Theses Citation Index* e *Google Scholar*, abrangendo o período supracitado. Durante a busca dos materiais foram identificados 129 textos científicos, todos internacionais, que abordavam a presença de material particulado de plástico em vários tipos de alimentos. No presente estudo, os autores enfatizaram a contaminação humana por alimentos marinhos, que podem ser consumidos “*in natura*” e/ou enlatados-embalados, contendo micropartículas (MP) plásticas.

## RESULTADOS

Na sequência, dos 129 estudos encontrados, 23 abordavam o consumo humano de animais marinhos com micropartículas plásticas e estão apresentados com letras variando de A até X (Quadro 1).

**Quadro 1:** Textos científicos envolvendo a temática de alimentos marinhos contaminados por partículas microplásticas e consumidos pelos humanos, 2017-2024 (n=23)

Grupos	Identificação/Título	Autores	País/Ano/ Fonte de Informação	Resumo	Delineamento do Estudo
**Moluscos**	A. The observation of starch digestion in blue mussel Mytilus galloprovincialis exposed to microplastic particles under varied food conditions	O’Brien CJ, Hong HC, Bryant EE, Connor KM	Estados Unidos 2021 PUBMED	Investigado o impacto dos microplásticos (MP) na atividade da enzima amilase de *Mytilus galloprovincialis* em condições controladas de laboratório. Identificou-se que a exposição aos níveis extremos de MP aumentou a atividade da amilase; entretanto, esses mexilhões podem ser resilientes aos atuais níveis de poluição por MP na natureza	Experimental
	B. An overview of microplastics in oysters: Analysis, hazards, and depuration	Liu Y, Shi H, Chen L, Teng X, Xue C, Li Z.	China 2023 PUBMED	Discutiu-se o impacto dos MP em ecossistemas marinhos, especialmente em ostras e destacou-se a necessidade de métodos analíticos padronizados para a sua quantificação em mariscos	Revisão
	C. The impact of nano/micro-plastics toxicity on seafood quality and human health: facts and gaps	Gündogdu, S, Rathod N, Hassoun A, Jamroz E, Kulawik P, Gokbulut C, Özogul F.	Peru Índia Polônia França 2023 PUBMED	A poluição ambiental e a consequente contaminação dos produtos do mar com MP representam uma ameaça potencial para os consumidores, estando o perigo mais elevado associado às algas, ao sal, aos mariscos e aos pequenos peixes	Revisão
**CRUSTÁCEOS**	D. Estimation of contamination level in microplastic-exposed crayfish by laser confocal micro-Raman imaging	Xiao X, Liu X, Mei T, Xu M, Lu Z, Dai H, *et al*.	China 2023 PUBMED	Mostrou-se os níveis de contaminação por MP em lagostins expostos às diferentes concentrações de MP e durações de exposição, utilizando técnicas de imagem por Laser Confocal Micro-Raman (LCM-Raman) e microscopia eletrônica de varredura. A imagem LCM-Raman combinada com a tecnologia de processamento de imagem pode ser usada para construir uma estratégia analítica de alto desempenho para a avaliação da contaminação por microplásticos em lagostins	Experimental
	E. Microplastics in feed cause sublethal changes in the intestinal microbiota and a non-specific immune response indicator of the freshwater crayfish Procambarus clarkii (Decapoda: Cambaridae)	Guillén-Watson R, Arias-Andres M, Rojas-Jimenez K, Wehrtmann IS.	Costa Rica 2023 PUBMED	MP são poluentes perigosos de preocupação global que ameaçam os ecossistemas aquáticos e a saúde pública e alteram a composição microbiana intestinal de P. clarkii. A exposição aos MP reduziu a abundância de Clostridia e Bateroidetes, importantes para o desenvolvimento do sistema imunológico e prevenção de patógenos	Experimental
	F. Progress on the Effects of Microplastics on Aquatic Crustaceans: A Review	Zhang S, Wu H, Hou J.	China 2023 PUBMED	Compreendeu-se os efeitos dos MP nos crustáceos aquáticos, fornecendo uma estrutura básica para a ameaça ecológica dos MP para esses crustáceos	Revisão
	G. Microplastic-mediated transport of PCBs? A depuration study with Daphnia magna	Gerdes Z, Ogonowski M, Nybom I, Ek C, Adolfsson-Erici M, Barth A, *et al*.	Suécia 2019 EMBASE	Avaliou-se o efeito do MP na remoção de bifenilas policloradas (PCB) em animais planctônicos expondo o cladócero *Daphnia magna* com uma alta carga corporal de PCB (18, 40, 128 e 209) à uma mistura de microplásticos e algas; os *daphnias* expostos apenas às algas serviram como controle. Houve maior produção de ovos específicos por tamanho nos animais portadores de PCB e que receberam alimentos misturados com MP. No entanto, os efeitos da exposição aos MP na fecundidade foram de baixa significância biológica, pois a carga corporal de PCB e as concentrações de exposição foram muito superiores às concentrações ambientais relevantes	Experimental
	H. Can microplastics influence the accumulation of pb in tissues of blue crab?	Munuera P, Salvat-Leal I, Belmonte A, Romero D	Espanha 2021 PUBMED	Avaliou-se a distribuição e acumulação de Pb nos tecidos de caranguejos azuis (*Callinectes sapidus*) expostos aos MP. A presença de MP na água não pareceu aumentar o acúmulo de Pb nos tecidos do caranguejo azul	Experimental
**PEIXES**	I. Reduced cellular process and developmental process genotoxicity of polystyrene nanoplastics in zebrafish embryogenesis using Aurelia aurita proteins	Park IH, Geum SW, Yeo M-K.	Coreia 2023 PUBMED	Embriões de peixe-zebra foram expostos aos nanoplásticos de poliestireno (PS-NP), mucina (Mu) e Mu + PS-NP para verificar seus efeitos na genotoxicidade. Com base nas anomalias fenotípicas em peixes jovens, a sequenciação de mRNA foi empregada para determinar as alterações nos níveis de transcrição gênica induzidas pelo PS-NP. O PS-NP induziu alterações na expressão de mRNA e teve efeitos tóxicos causadores de anomalias fenotípicas nos embriões de peixe-zebra	Experimental
	J. Assessment of microplastic contamination in an eastern Pacific tuna (Katsuwonus pelamis) and evaluation of its health risk implication through molecular docking and metabolomics studies	Wu L, Dai X, Xu J, Ou D, Wang L, Lin H, *et al*	China 2023 PUBMED	Foi investigada a poluição por MP em uma espécie de atum comercialmente importante, *Katsuwonus pelamis* do Pacífico Oriental e suas implicações para a saúde. Foi analisada a presença de MP em várias partes do peixe, identificando os tipos predominantes de MP, como poliéster e tereftalato de polietileno (PET) e examinadas suas interações moleculares com a proteína glicerol quinase	Experimental
	K. Natural history matters: Plastics in estuarine fish and sediments at the mouth of an urban watershed	Talley TS, Venuti N, Whelan R.	Estados Unidos 2020 EMBASE	Foi examinado um trecho do córrego Chollas Creek em San Diego, Califórnia, para determinar: a extensão e a magnitude da poluição por microplásticos em sedimentos estuarinos e peixes, a extensão e magnitude da contaminação por SVOC (Compostos Orgânicos Voláteis Semi-Voláteis) nos peixes e se eles ingeriam quais tipos de MP, em comparação com a composição de MP dos sedimentos do leito do córrego. Os sedimentos (0-5 cm de profundidade) continham cerca de 10.000 pequenos pedaços de plástico por m^2^, consistindo 90% de fibras e peças duras e moles. Quase 25% dos peixes continham pequenos plásticos; a prevalência variou com o tamanho e entre espécies; dos 25 tipos de plásticos encontrados nos sedimentos, os peixes preferiram cerca de 10 tipos; vários SVOC, tanto compostos solúveis em água quanto associados aos sedimentos, foram encontrados nas duas espécies de peixes testadas	Experimental
	L. Presence of microplastics in commercial canned tuna	Diaz-Basantes MF, Nacimba-Aguirre D, Conesa JA, Fullana A	Equador Espanha 2022 Elsevier	Determinou-se a presença de micropartículas poliméricas sintéticas em amostras de atum enlatado. A identificação dos MP por fluorescência e microespectrometria FTIR revelou que PET, poliestireno e nylon foram os mais presentes nas amostras analisadas	Experimental
	M. No evidence of spherical microplastics (10-300 μm) translocation in adult rainbow trout (Oncorhynchus mykiss) after a two-week dietary exposure	Kim J, Poirier D. G, Helm P. A, Bayoumi M, Rochman C. M.	Canadá 2020 PUBMED	Descobriu-se nas trutas que os MP são translocados do intestino para outros tecidos, tornando uma preocupação potencial à exposição ao comer filés de peixe ou outros produtos do mar. Mas não foi encontrada partícula nos filés, fígado ou gônadas dos peixes, sugerindo que a translocação de MP esféricos não ocorreu em trutas arco-íris adultas	Experimental
	N. The effect of environmental stressors on growth in fish and its endocrine control	Canosa LF, Bertucci JI.	Argentina Espanha 2023 PUBMED	Obteve-se uma visão geral e atualização sobre os efeitos de vários fatores antropogênicos abióticos sobre o hormônio do crescimento e a regulação da ingestão alimentar. MP podem ser ingeridos pelos peixes e acumulados no estômago, gerando uma sensação de saciedade ou danos que podem inibir a ingestão de alimentos; seu acúmulo no estômago de peixes pode alterar a resposta de fatores periféricos que regulam a ingestão de alimentos	Revisão
**FRUTOS DO MAR**	O. Bioaccumulation and biomagnification of microplastics in marine organisms: A review and meta-analysis of current data	Miller ME, Hamann M, Kroon FJ.	Austrália 2020 PUBMED	A contaminação por MP no ambiente marinho é um problema crescente. Estudos anteriores demonstraram que os MP bioacumulam-se em organismos marinhos, mas a biomagnificação desses compostos ao longo da cadeia alimentar ainda é debatido. Esta revisão sistemática da literatura não encontrou evidências claras de biomagnificação de MP em uma teia alimentar marinha geral, embora a bioacumulação de MP ocorra dentro dos níveis tróficos. A falta de dados sobre a transferência trófica e biomagnificação de aditivos químicos associados aos MP é um gap a ser preenchido futuramente	Revisão sistemática com meta-análise
**FRUTOS DO MAR**	P. Microplastics in seafood: How much are people eating?	Wendee Nicole	Estados Unidos 2021 PUBMED	Quantificada a presença de MP em diversas espécies marinhas, incluindo peixes e mariscos, indicando que essas partículas estão amplamente distribuídas no ambiente maritimo. O consumo regular de frutos do mar pode levar à ingestão acumulativa de MP, levantando preocupações sobre os efeitos potenciais à saúde, a longo prazo	Teórico-reflexivo
	Q. Microplastics as a new, ubiquitous pollutant: Strategies to anticipate management and advise seafood consumers	Susan E. Farady	Estados Unidos 2019 SCIENCE DIRECT	A presença de MP nos oceanos é crescente, afetando a vida marinha e potencialmente a saúde humana pelo consumo de frutos do mar. A sua ingestão por organismos marinhos pode ter impactos adversos na saúde dos consumidores. Sugere-se a implementação de estratégias para gerenciar e mitigar a poluição por MP, incluindo políticas de redução e conscientização dos consumidores	Teórico-reflexivo
	R. - Investigating microplastics bioaccumulation and biomagnification in seafood from the Persian Gulf: a threat to human health?	Razegheh Akhbarizadeh, Farid Moore, Behnam Keshavarzi	Irã 2019 PUBMED	Foi investigada a presença de MP em cinco espécies de frutos do mar comerciais. Os MP estavam presentes em todos os espécimes, com níveis mais altos encontrados em músculos e brânquias de espécies escavadoras e filtradoras. No entanto, não se encontrou evidências de biomagnificação de MP nas partes comestíveis dos frutos do mar, sugerindo que ocorre diluição trófica em vez de magnificação. Então, é importante controlar o consumo de frutos do mar para grupos vulneráveis, como mulheres grávidas ou lactantes e seus filhos, para garantir sua segurança	Experimental
	S. Microplastics in fisheries and aquaculture: status of knowledge on their occurrence and implications for aquatic organisms and food safety	Lusher A, Hollman P, Mendoza-Hill J	Reino Unido Holanda Espanha Itália2017 PUBMED	O aumento da produção de plásticos levou a uma contaminação significativa dos ambientes aquáticos. Os MP podem ser ingeridos por organismos aquáticos e acumular substâncias químicas tóxicas. No entanto, estudos mostraram que a ingestão de microplásticos por humanos é reduzida pela remoção do trato gastrointestinal na maioria das espécies de frutos do mar consumidos. Além disso, a exposição a microplásticos após o consumo de frutos do mar é considerada negligenciável	Revisão
	T. Microplastic contamination of seafood intended for human consumption: A systematic review and meta-analysis	Danopoulos E, Jenner LC, Twiddy M, Rotchell JM.	Reino Unido 2020 PUBMED	Realizou-se uma revisão sistemática e meta-análise dos níveis de contaminação por MP em frutos do mar e, posteriormente, estimou-se a absorção humana anual. Foram encontrados microplásticos nas amostras advindas de frutos do mar que se constituem em importantes vetores para a exposição humana aos MP	Revisão sistemática com meta-análise
	U. Vibrio parahaemolyticus and Vibrio vulnificus in vitro biofilm dispersal from microplastics influenced by simulated human environment	Leighton RE, Xiong L, Anderson GK, Astarita GM, Cai G, Norman RS, *et al*	Estados Unidos 2023 PUBMED	A ingestão de microplásticos contaminados com biofilmes de Vibrio pode ser um risco para a saúde humana. Estudo investigou como diferentes condições ambientais afetam a dispersão de biofilmes de *Vibrio parahaemolyticus* e *Vibrio vulnificus* em MP. Os resultados mostraram que ambas as espécies podem se dispersar rapidamente em diferentes tipos de plásticos, mas o processo de dispersão é altamente variável e dependente do tipo de plástico e das condições ambientais. Isso sugere que a ingestão de MP contaminados pode ser um risco para a saúde humana, especialmente em áreas com sua alta concentração	Experimental
	V. Microplastics: A Real Global Threat for Environment and Food Safety: A State of the Art Review	Ziani K, Ioniţă-Mîndrican C-B, Mititelu M, Neacşu SM, Negrei C, Moroşan E, *et al.*	Romênia 2023 PUBMED	Analisou-se a presença generalizada de MP no meio ambiente e seu impacto na vida selvagem e na saúde humana. Discutiu-se a sua detecção em várias espécies marinhas, água potável e alimentos comuns, como sal, mel e organismos marinhos	Revisão
	X. Uncovering microplastics contamination in canned seafood	Silva DM, Almeida CMR, Guardiola FA, Pereira R, Rodrigues SM, Ramos S.	Portugal e Espanha 2024 Elsevier	Foram investigadas 60 latas de frutos do mar (sardinha em molho de tomate, em óleo de girassol, polvo em molho de tomate, mexilhões em molho de escabeche, atum em azeite e cavala em óleo de girassol) de 4 marcas populares de Portugal e Espanha. Todos os tipos de frutos do mar enlatados investigados estavam contaminados por MP	Experimental

## DISCUSSÃO

Os pesquisadores que realizaram estes estudos foram europeus, norte-americanos, asiáticos, da Oceania, da América Central e da América do Sul, demonstrando o problema global dos MP em animais marinhos consumidos pelos homens. O idioma de todos os estudos foi o inglês; a maior parte envolveu pesquisadores norte-americanos; a fonte de informação em que mais foram encontrados foi a PUBMED; a maioria dos estudos foi feita não isoladamente, mas por grupos de pesquisadores e alguns envolveram estudiosos de vários países, simultaneamente.

Evidenciou-se que a poluição mundial de material particulado nos mares é um grave problema, que contradiz os dados projetados pela ONU em relação aos ODS da Agenda 2030, ou seja: até 2030, em relação à saúde e bem-estar, deveria estar assegurado uma vida saudável e o bem-estar precisaria ser promovido para todos(as), em todas as idades; além disso, necessitaria haver uma redução da poluição/contaminação ambiental; os países precisariam reforçar a redução de riscos e o gerenciamento de riscos nacionais e globais de saúde, conforme preconizado na Agenda 2030, citada anteriormente. Entretanto, ao contrário, água, solo e ar estão poluídos e provocando severos transtornos nos seres vivos. Estranha-se, também, a incipiência da atuação dos profissionais de saúde em relação a esta problemática, parecendo haver certo desconhecimento sobre o assunto, embora existam importantes pesquisas sobre o tema, conforme mostrado nos textos analisados nesta revisão.

Quanto a contaminação dos alimentos marinhos, identificou-se que os MP foram encontrados nos moluscos do tipo mexilhões, ostras, algas, mariscos e outros pequenos peixes (textos “A”-“C”). No Brasil, estima-se que organismos marinhos estão ingerindo plásticos, desde zooplâncton, peixes e tartarugas, mamíferos e aves marinhas, muitos deles já ameaçados de extinção. Tais espécies marinhas estão tendo contato com resíduos da produção humana e, também, morrendo devido a eles^(11)^.

Em relação aos crustáceos, identificou-se a contaminação de lagostins (textos D-E) e, chumbo em caranguejos (texto H) consumidos pelos homens. No Brasil, foi identificado o quanto a poluição afetou a vida dos caranguejos-uçá dos manguezais de cidades litorâneas, submetidos à grande quantidade de metais pesados encontrados nas águas e ingeridos pelos brasileiros, arriscando a saúde destes consumidores^(12)^.

Quanto aos peixes, foram várias as pesquisas realizadas mostrando que a poluição por MP de poliestireno foi genotóxica em embriões de peixes-zebra na Coreia (texto “I”); contaminou os atuns na China (texto “J”) e foi encontrada também por pesquisadores espanhóis e equatorianos em atuns comumente comercializados (artigo “l). Nos frutos do mar, mostrou-se contaminante também em mariscos e peixes, indicando que este material particulado pode causar danos à saúde a longo prazo (textos “P”, “Q”) em estudos realizados no Golfo Pérsico (“R”), bem como em demais países asiáticos e europeus. Frutos do mar enlatados apresentam, igualmente, importante contaminação, tal como encontrado em Portugal e na Espanha, em todas as latas examinadas (texto “X”).

Observa-se que apesar do Brasil ter uma extensão litorânea importante, possivelmente muito maior do que muitos dos países identificados neste texto reflexivo, não foram encontrados estudos sobre este tema, em especial no período investigado e, tampouco, constatou-se a presença de profissionais de saúde neste contexto. Falhas no sistema de gestão de resíduos têm sido as responsabilizadas pelo problema da poluição plástica; a culpabilidade costuma centrar-se no consumidor (que descarta estes materiais inadequadamente) e nas cidades, sem coletas seletivas e sistemas de reciclagens. Soluções políticas têm se concentrado em melhorar a reciclabilidade e as taxas de reciclagem dos produtos e embalagens de plástico^(11)^.

No cenário de busca para o alcance do desenvolvimento sustentável, os profissionais da saúde deveriam ter uma participação vital para o êxito em tais metas e objetivos. O ODS 3 é dedicado à ‘Saúde e ao Bem-Estar’ e, em sua composição, há 9 metas extremamente complexas e desafiantes para toda a sociedade. Destaca-se aqui a meta de reduzir o número de mortes e doenças por produtos químicos perigosos, contaminação e poluição do ar e água do solo^(13)^, o que mereceria o envolvimento de ações mais assertivas por parte de todos, incluindo os do setor saúde.

## CONSIDERAÇÕES FINAIS

Mediante as colocações no presente estudo, as apresentações dos textos científicos e as reflexões realizadas, destaca-se a relação da exposição e contaminação humana pelas MP presentes em alimentos marinhos consumidos pela população e que podem trazer danos à saúde. Faz-se necessário a realização de estudos futuros que possam demonstrar, com mais profundidade, os danos que tais MP podem causar no organismo das pessoas. Com isso, é importante a criação de maior número de políticas públicas nacionais direcionadas à prevenção da poluição das águas, assim como, proporcionar a orientação da equipe de saúde sobre os danos que alimentos com MP podem causar, para ser possível uma maior aproximação do país às metas de ação dos ODS.

Espera-se que este texto possa alertar os profissionais de saúde para que realizem atendimentos e orientações aos usuários dos serviços de saúde, sobre este tema.
